# Supplementary material for: Double-slit photoelectron interference in strong-field ionization of the neon dimer
Source: Nat Commun. 2019 Jan 2;10:1. doi: 10.1038/s41467-018-07882-8 (PMC6315036; doi:10.1038/s41467-018-07882-8)
Supplement: Supplementary file 1 — Supplementary Information [file 41467_2018_7882_MOESM1_ESM.pdf]

Supplementary Information

Double-slit photoelectron interference in strong-field ionization of the neon dimer

Maksim Kunitski, Nicolas Eicke, Pia Huber, Jonas Köhler, Stefan Zeller, Jörg Voigtsberger, Nikolai Schlott, Kevin Henrichs, Hendrik Sann, Florian Trinter, Lothar Ph. H. Schmidt, Anton Kalinin, Markus S. Schöffler, Till Jahnke, Manfred Lein and Reinhard Dörner

Contents

Supplementary Figures 2

Supplementary Figure 1. Sketch of the experimental setup. 2

Supplementary Figure 2. Dependence of the dissociation time on the initial internuclear distance. 3

Supplementary Figure 3. Photoelectron momentum distributions from advanced theory. 3

Supplementary Figure 4. Photoelectron spectra in the natural molecular frame. 4

Supplementary Figure 5. Fits of interference profiles with the corresponding residuals. 4

Supplementary Figure 6. The momentum and energy distributions of Ne^+ 5

Supplementary Figure 7. The molecular frame photoelectron momentum distribution of the background. 5

Supplementary Figure 8. Background influence on the interference fringe contrast. 6

Supplementary Figure 9. Estimation of the interference fringe contrast. 7

Supplementary Notes 8

Supplementary Note 1. Dissociation times 8

Supplementary Note 2. Advanced theory: active electron coupled to a two-level ion 9

Supplementary Note 3. Molecular-frame transformation 11

Supplementary Note 4. Determination of the bond length 12

From the interference pattern 12

From ion momentum by potential mapping 12

Supplementary Note 5. False coincidences 13

Supplementary Note 6. The interference fringe contrast 14

Supplementary References 15

Supplementary Figures

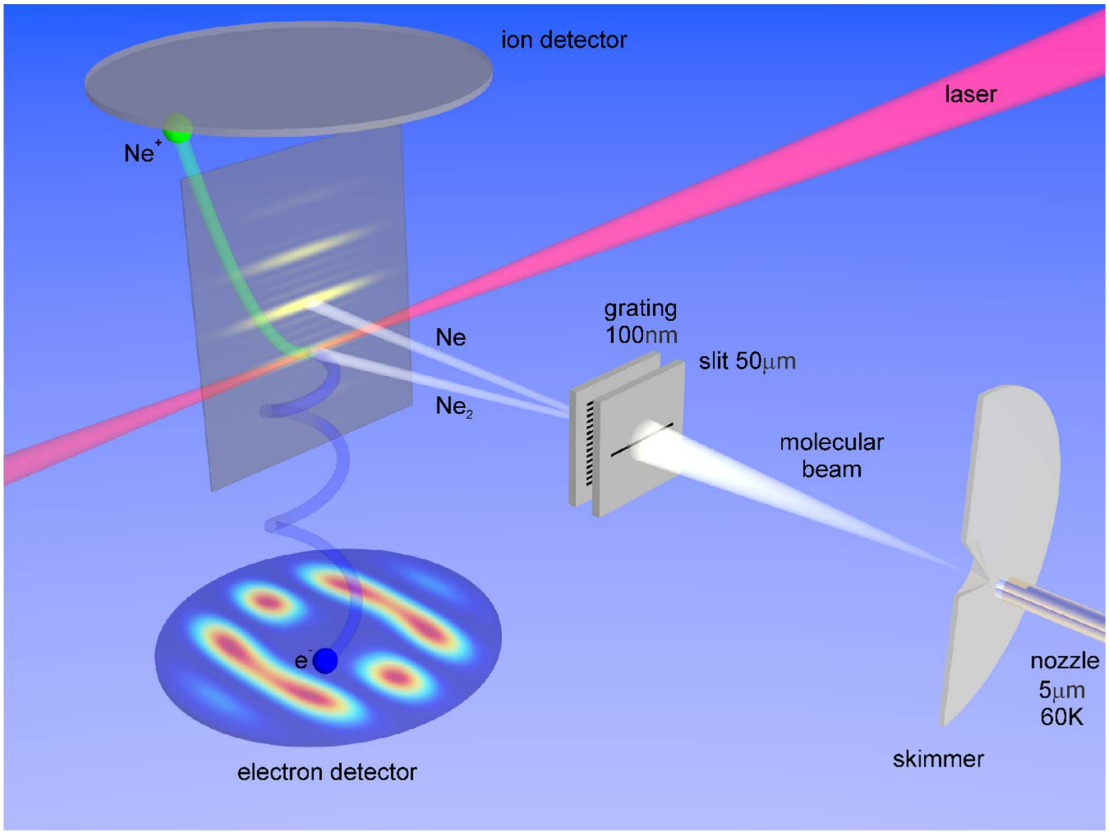

Supplementary Figure 1. Sketch of the experimental setup. COLTRIMS meets matter wave diffraction. Neon dimers are produced in the supersonic expansion of the neon gas through 5 µm nozzle into the vacuum. The transmission grating with a period of 100 nm is used to deflect neon dimers towards the laser focus, where dimers get ionized. The electron and the neon ion are guided by weak electric and magnetic fields towards two position sensitive detectors. Measuring the impact position as well as time a charged particle needs to reach the detector allows to deduce the 3D momentum of a particle after ionization.

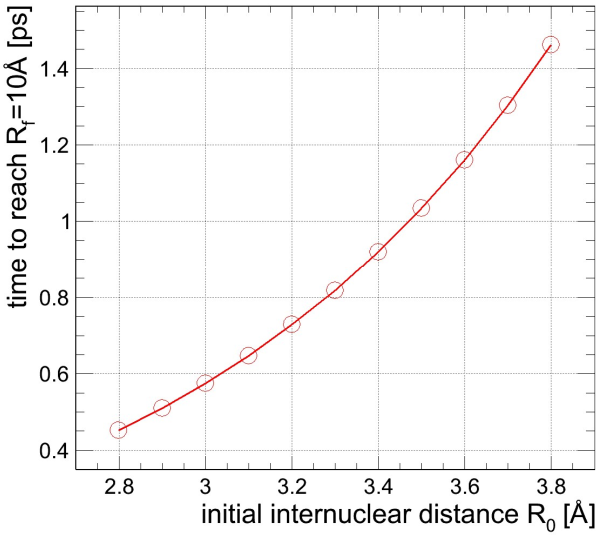

Supplementary Figure 2. Dependence of the dissociation time on the initial internuclear distance. The time the neon dimer needs to reach an internuclear distance of 10 Å during direct dissociation along the $II(1/2)_g$ potential curve.

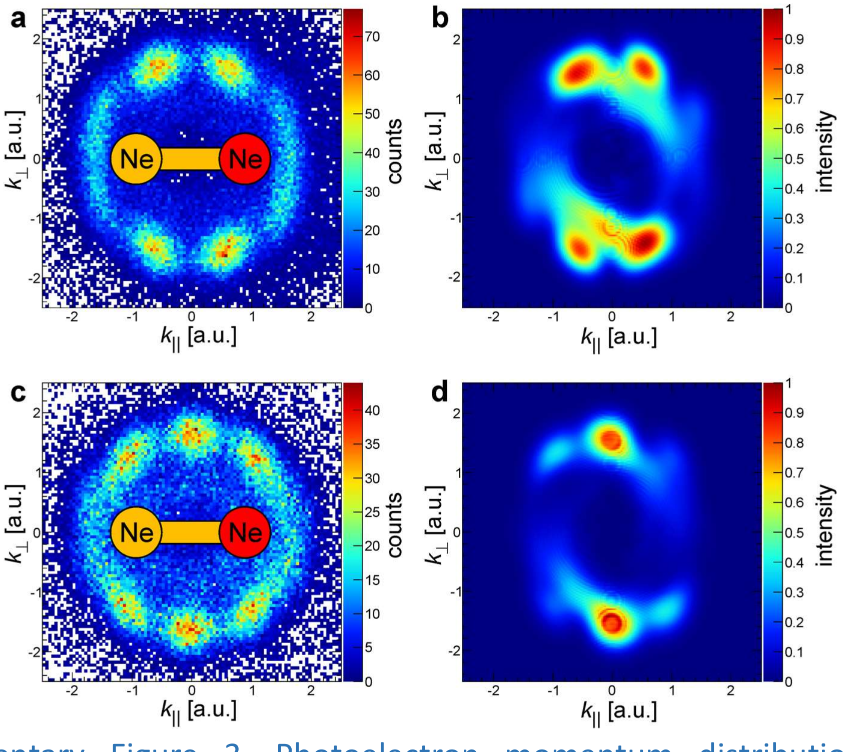

Supplementary Figure 3. Photoelectron momentum distributions from advanced theory. Photoelectron momentum distributions in the molecular frame: **a** and **c** – measured; **b** and **d** – theory with an active electron coupled to a two-level ion. Upper panels: *gerade* channel; lower panels: *ungerade* channel. The red side of the sketched molecule defines the momentum direction of the measured neon ion.

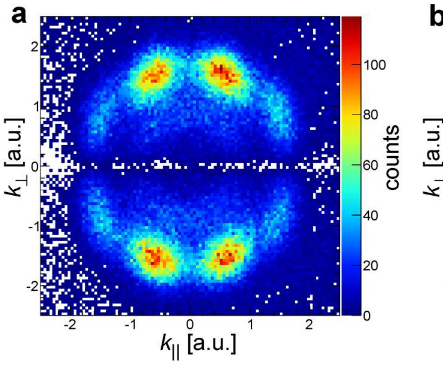

Supplementary Figure 4. Photoelectron spectra in the natural molecular frame. **a** - the direct dissociation channel; **b** - the indirect dissociation channel.

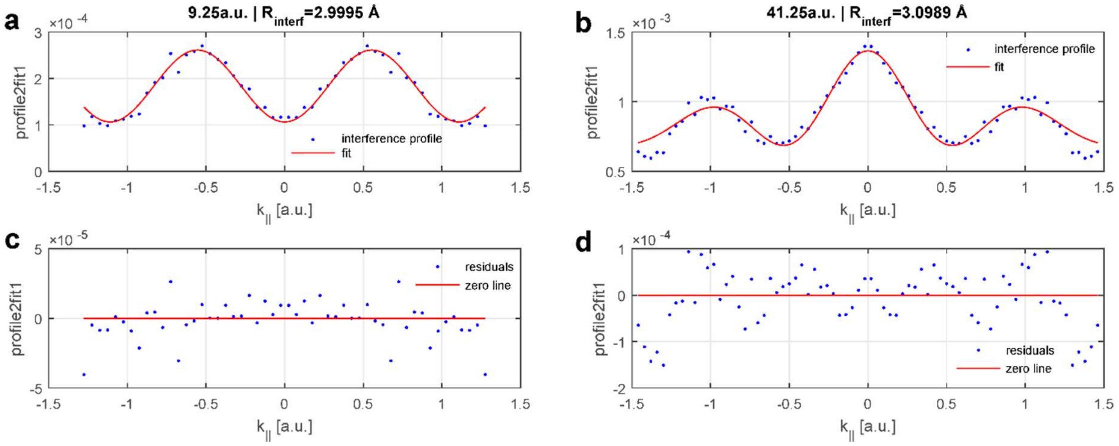

Supplementary Figure 5. Fits of interference profiles with the corresponding residuals. **a**: the interference profile of the direct fragmentation channel at an ion momentum of 9.25 a.u. corresponds to a bond length of 3.00 Å; **b**: the interference profile of the indirect fragmentation channel at an ion momentum of 41.25 a.u. corresponds to a bond length of 3.10 Å. Profiles are from Fig. 4a,c. **c** and **d** are residuals from the fits of the interference profiles in **a** and **b**, respectively.

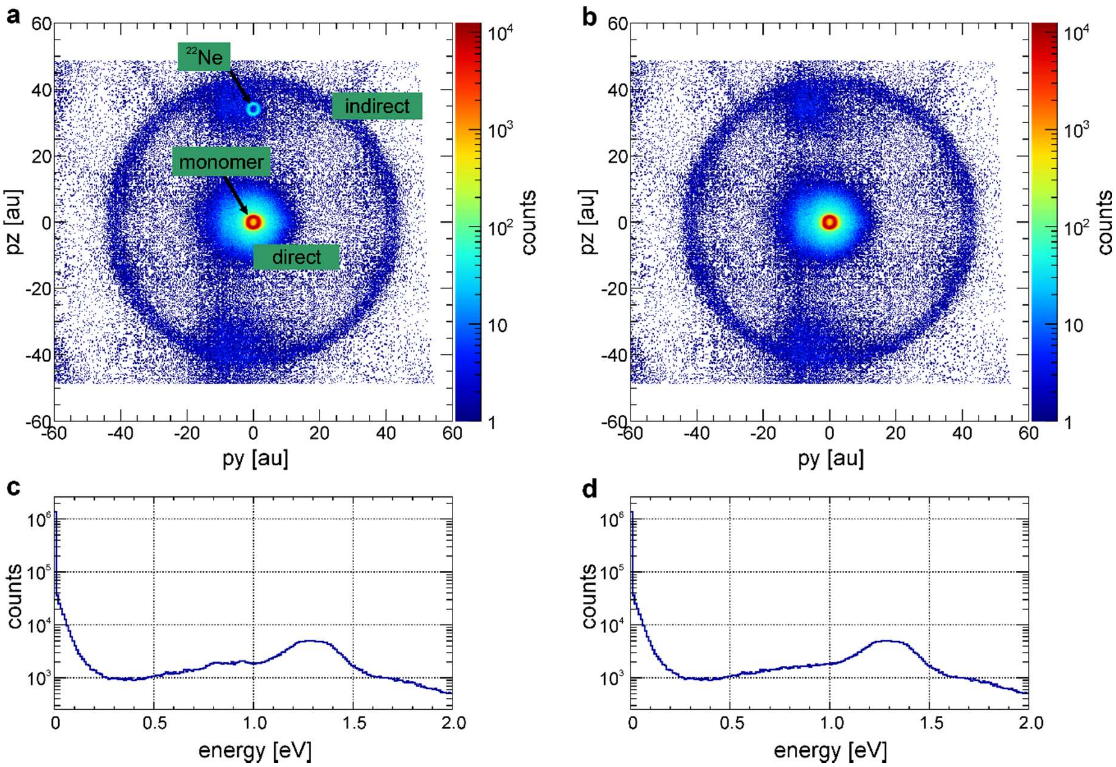

Supplementary Figure 6. The momentum and energy distributions of Ne^{+}. **a** and **c** – as it was measured in the experiment, **b** and **d** – after removing events corresponding to the ^{22}Ne single ionization.

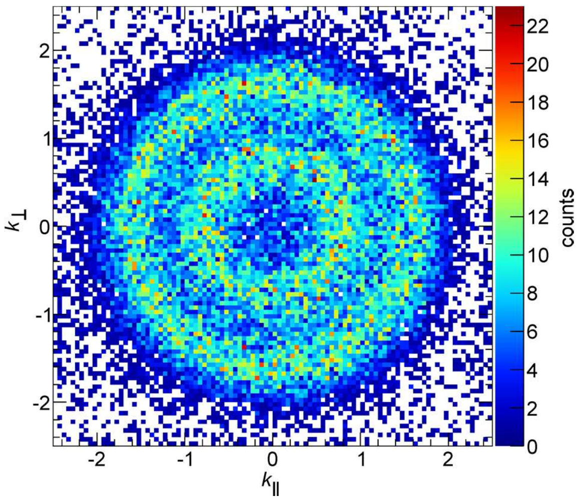

Supplementary Figure 7. The molecular frame photoelectron momentum distribution of the background. As the background, the ion momentum magnitude has been chosen to be higher than 18 a.u. but lower than 35 a.u.

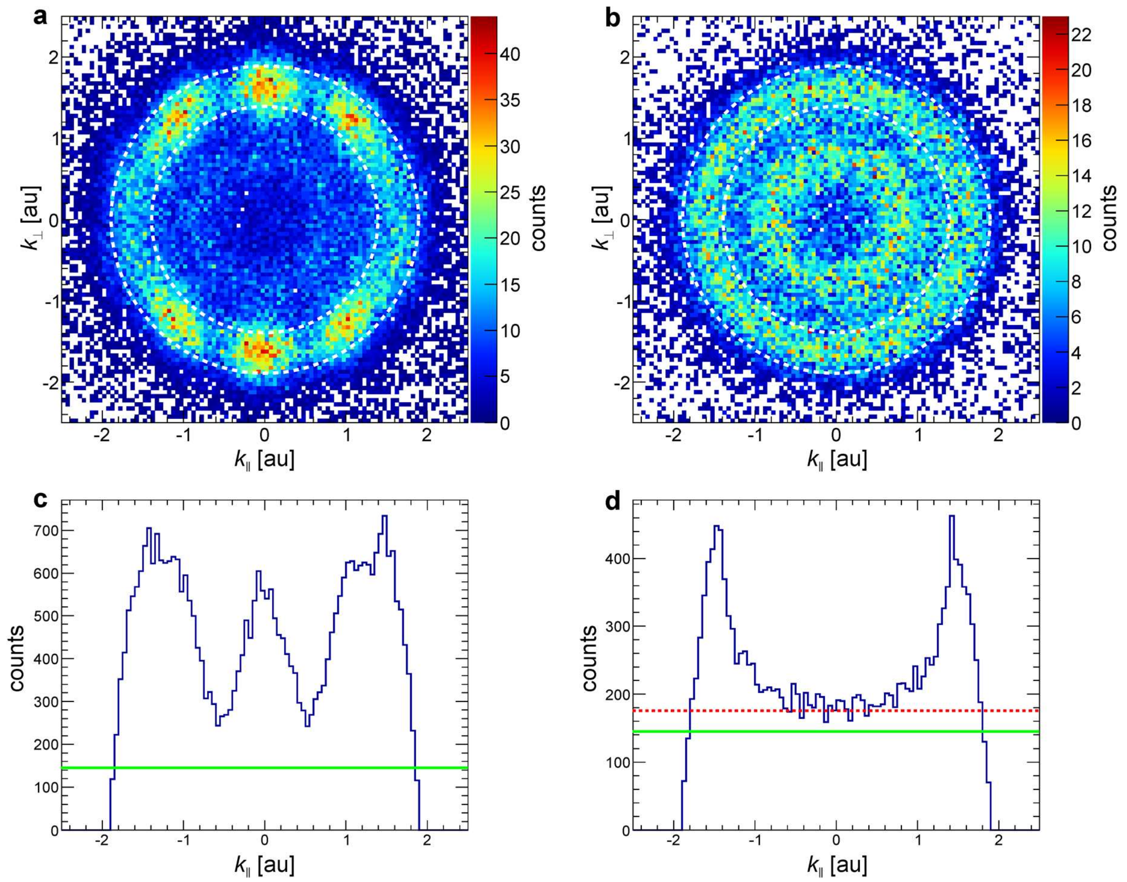

Supplementary Figure 8. Background influence on the interference fringe contrast. The photoelectron momentum distributions in the molecular frame (**a** and **b**) and the corresponding $k_\parallel$-projections (**c** and **d**) of the region between two dashed lines (1.4-1.9 a.u.). **a** and **c** - indirect breakup channel with ion momenta between 37 a.u. and 46 a.u. **b** and **d** - the background (false coincidences) with ion momenta between 18 a.u. and 35 a.u. In addition, the following cuts have been applied: $|p_x|<0.55$ a.u. for electrons and $|p_x|<12.0$ a.u. for ions. The volume of the indirect break up in the momentum space of the ion is by 0.83 times smaller than that of the chosen background region. The green line is the estimated background signal caused by false coincidences.

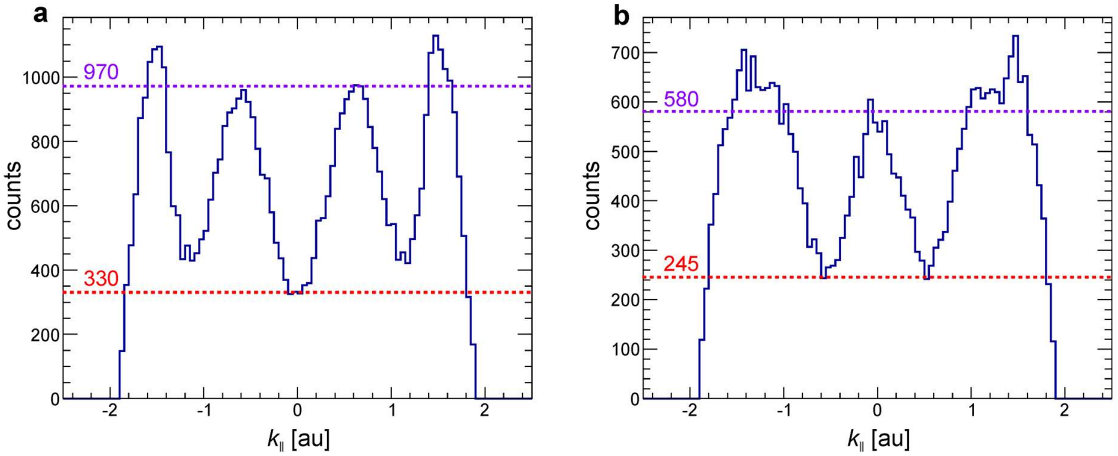

Supplementary Figure 9. Estimation of the interference fringe contrast. The $k_{\parallel}$-projections of the photoelectron momentum distribution in the molecular frame for an electron momentum region of 1.4-1.9 a.u., as shown in Supplementary Figure 8. **a** – the direct fragmentation channel, **b** – the indirect fragmentation channel.

Supplementary Notes

Supplementary Note 1. Dissociation times

We have estimated dissociation times by classical propagation of a particle with reduced mass of Ne_2 (μ=10 amu) along the potential curve $II(1/2)_g$ from ref. [1]. The times the dimer needs to reach an internuclear distance of 10 Å (taken as a reference) during dissociation starting at different internuclear distances according to the ground state probability distribution are shown in Supplementary Figure 2.

Thus, in the worst case, when dimer starts to dissociate along $II(1/2)_g$ at an internuclear distance of 3.8 Å, it takes 1.5 ps to reach 10 Å. The rotational times, however, are much longer: $T_{\mathrm{rot}} \sim \frac{1}{2B} = \mu R^2$, where $B = \frac{1}{2I}$ is the rotational constant in atomic units, and $I = \mu R^2$ is the moment of inertia. For Ar_2 with μ=20.0 amu and R=3.83 Å, T_{rot}=290 ps (see ref. PRA, 83, 061403(R), 2011). Given μ=10.0 amu and R=3.2 Å for Ne_2, $T_{\mathrm{rot}} \sim$100 ps.

According to our estimations, the indirect dissociation happens even faster: an internuclear distance of 10 Å is reached within ca. 200-300 fs. Qualitative explanation for this is that the initial movement on the $I(1/2)_u$ potential should be accomplished within a laser pulse (40 fs, FWHM in intensity), otherwise the vibrational wave packet will not be lifted up to the dissociative $II(1/2)_g$ potential curve, leaving dimer ion bound. The subsequent movement on the $II(1/2)_g$ potential curve is faster than in case of direct dissociation, since the starting internuclear distance is much shorter. The same dissociation path was used for explanation of the 200 fs-long signal depletion in the pump-probe experiment with Ar_2[2]. Since the potential energy curves of Ne_2 are similar to those of Ar_2, but the reduced mass is only a half of Ar_2, the dissociation dynamics of Ne_2^+ should be even faster.

Supplementary Note 2. Advanced theory: active electron coupled to a two-level ion

The purpose of this section is a further theoretical investigation of Ne_2 strong-field ionization using an extended model which allows us to include the hole dynamics in the remaining ion and its interaction with the laser field as well as the correlated interaction of the outgoing electron with the parent ion.

We describe the Ne_2^+ molecular ion as a two-level system where the states $(1,0)$ and $(0,1)$ correspond to the hole being located at the neon atom on the negative or positive x-axis, respectively. The ionic Hamiltonian is

$$H_{\mathrm{ion}} = \begin{pmatrix} E_x(t)\cdot x_0 & \alpha \\ \alpha & -E_x(t)\cdot x_0 \end{pmatrix},$$

where $x_0 = R/2 = 2.93$ a.u. is half the equilibrium distance between the two neon atoms in the neutral dimer and $2\alpha = 0.00870$ a.u. is tuned to obtain the energy splitting between the *gerade* and *ungerade* states of the molecular ion at this distance. An active electron is coupled to the two-level system such that a state of the complete system is written as a vector of two wave functions, $\Psi(t) = (\psi_{\mathrm{L}}(\mathbf{r},t), \psi_{\mathrm{R}}(\mathbf{r},t))$, undergoing coupled dynamics prescribed by the time-dependent Schrödinger equation (TDSE) $i\partial_t\Psi = H\Psi$ with the Hamiltonian $H = H_{\mathrm{ion}} + T + V$, where

$$T = \frac{1}{2}\left(-i\vec{\nabla} + \mathbf{A}(t)\right)^2, \qquad V = \begin{pmatrix} V_{\mathrm{L}}(\mathbf{r}) & 0 \\ 0 & V_{\mathrm{R}}(\mathbf{r}) \end{pmatrix}.$$

This allows us to use two different potentials, $V_{\mathrm{L}}(\mathbf{r})$ and $V_{\mathrm{R}}(\mathbf{r})$, depending on the charge distribution in the ion. Each of the potentials consists of two different parts, one describing the interaction of the electron with a Ne^+ ion and another one describing the interaction with a neutral Ne atom. In the case of V_{L}, the ion is placed on the negative x-axis and the neutral atom on the positive x-axis, i.e. $V_{\mathrm{L}}(\mathbf{r}) = V_{\mathrm{ion}}(\mathbf{r} + x_0\mathbf{e}_x) + V_{\mathrm{atom}}(\mathbf{r} - x_0\mathbf{e}_x)$. For the electron-ion interaction we use the same potential as in the main text. The interaction with the neutral neon atom is given by a neutral-neon scattering potential[3]. The weak energy dependence of the latter potential is neglected and the potential is evaluated at zero energy. We follow a similar pseudopotential procedure as in the main text and remove the singularity by matching to an s-wave scattering state at momentum $p = 1$ a.u. at a distance 1 a.u. from the singularity.

The initial state is an eigenstate of the field-free Hamiltonian and consists of two *p*-type orbitals for ψ_{L} and ψ_{R} located at the respective ions. It has total *gerade* symmetry, where the symmetry operation in the coupled system inverts the coordinates in the wave function of the active electron and exchanges the two states of the two-level system, i.e. $\psi_{\mathrm{L}}(\mathbf{r}) \leftrightarrow \psi_{\mathrm{R}}(-\mathbf{r})$. Starting from this bound state, the TDSE is solved with the split-operator method. Here, the short-time propagator is split as

$$e^{-i\,dt\,H} = e^{-i\,dt\,V/2} e^{-i\,dt\,(H_{\mathrm{ion}}+T)} e^{-i\,dt\,V/2} + O(dt^3),$$

and further

$$e^{-i\,dt\,(H_{\mathrm{ion}}+T)} = e^{-i\,dt\,H_{\mathrm{ion}}} e^{-i\,dt\,T}.$$

All parameters are chosen as in the main text. We also account for the different possible orientations of the dimer with respect to the polarization plane. After the time evolution, the final state is projected onto the *gerade* and *ungerade* states of the molecular ion, leading to

$$\psi_{\pm} = \frac{1}{\sqrt{2}}(\psi_{\mathrm{R}} \pm \psi_{\mathrm{L}}).$$

From these single-electron wave functions we obtain the photoelectron momentum distributions for the two types of interference patterns shown in Supplementary Figure 3. In contrast to the calculations from the main text that had perfect contrast by construction, these distributions show a significant decrease in contrast. In the presence of a second atom in the case of the dimer, the two pathways in strong-field ionization are not completely equivalent anymore. This is different from XUV photoionization where perfect contrast has been observed[4].

Another source that reduces the contrast are false coincidences (see Supplementary Note 4). However, they account for only one-half of the background in the case of the indirect dissociation, and are rather irrelevant for the direct breakup channel.

As seen in Supplementary Figure 3 the advance theory produces some additional features in the photoelectron momentum distribution that are not observed in the experiment. The reason for this might be an insufficient accuracy of the model potential for the neutral atom that was used in the TDSE calculations. The model potential for the neutral Ne atom is taken from Supplementary Reference 3. The quality of the potential in this reference was checked by comparing the computed electron scattering cross-sections based on the proposed potential with the experimental ones. This comparison was done however for electrons with energies higher than 50 eV (a momentum of 1.9 a.u.). Moreover, it was stated that the experimental scattering cross-section is not well reproduced by the simulation based on the model potential in the low energy range. Since electrons upon tunneling have even lower energies ($E<3.4$ eV or $p<0.5$ a.u.), as was measured from the experimental momentum distribution perpendicular to the polarization plane (where the laser field is zero), one might assume that the accuracy of the model potential in the desired electron energy region is not that high. Another possible source of error relates to the fact that the potential of the neutral atom had to be converted into a pseudopotential to make the calculation feasible.

The idea, however, behind using the advance theory was to give qualitative explanation of the finite contrast in the measured interference pattern.

Supplementary Note 3. Molecular-frame transformation

In the natural molecular-frame transformation, the ion momentum vector, not its projection to the polarization plane, defines the $k_{\parallel}$ direction. The electron momentum vector is then projected onto the $k_{\parallel}k_{\perp}$-plane to get the molecular frame photoelectron momentum distributions shown in Supplementary Figure 4. This projection conserves $\mathbf{k} \cdot \mathbf{R}$ but the momentum distributions show a node along the molecular axis due to the vanishing volume element.

Supplementary Note 4. Determination of the bond length

From the interference pattern

The accurate measurement of the bond length would require a more advanced theoretical model than the very simple one used for simulations in Fig. 4 b,d, since the ionization weighting discussed in the paper changes the shape of the interference fringes. We could partially reduce this weighting by dividing the experimental momentum distribution by the corresponding spectrum of the monomer, however, it was not possible to completely remove it. Moreover, the ionization process depends on internuclear distance due to change in the ionization potential. This dependence should be considered as well for accurate estimation of the bond length distribution.

Despite these arguments, we have done estimation of a bond length by fitting the fringe distributions corresponding to different ion momenta (Fig. 4a,c) to a function based on eq. (1) from the main text:

$$f(k_{\parallel}) = A \cdot cos^2\left(\frac{k_{\parallel} \cdot R_{\mathrm{interf}}}{2} + \frac{\Delta\phi}{2}\right) \cdot e^{-\frac{k_{\parallel}^2}{\sigma}} + B,$$

where R_{interf} is the internuclear distance, $\Delta\phi$ is a phase that is 0 for the indirect channel and π for the direct one, σ is the doubled variance of the residual Gaussian distribution (ionization weighting). The Gaussian distribution was used only for the indirect channel, since the direct one has only two fringes that are symmetric with respect to $k_{\parallel} = 0$. A and B are the amplitude and the background, respectively. The typical fits for the direct and indirect fragmentation are shown in Supplementary Figure 5.

From ion momentum by potential mapping

In order to obtain the bond length from the measured ion momenta we have inverted the $\mathrm{II}(1/2)_g$ and $\mathrm{I}(1/2)_u$ energy potentials for the direct and indirect dissociation channels, respectively. The potentials have been taken from ref. [1]. The initial potential energies (with respect to the dissociation energy of 21.6 eV) of the ion upon ionization have been calculated as following:

$$E_{\mathrm{d}} = \frac{p_{\mathrm{d}}^2}{m_{\mathrm{Ne}}},$$

$$E_{\mathrm{id}} = \frac{p_{\mathrm{id}}^2}{m_{\mathrm{Ne}}} - E_{\mathrm{ph}} + E_{\mathrm{so}},$$

where p_{d} and p_{id} are measured ion momenta for the direct and indirect dissociation channels, respectively. $E_{\mathrm{ph}} = 1.59\ eV$ is the photon energy corresponding to a central wavelength of 780 nm. $E_{\mathrm{so}} = 0.1\ eV$ is the spin-orbit coupling. After calculating the energies E_{d} and E_{id}, the corresponding internuclear distances R_{d} and R_{id} (both labeled as R_{ion} in Fig. 5) have been found from $\mathrm{II}(1/2)_g$ and $\mathrm{I}(1/2)_u$ energy potentials.

Supplementary Note 5. False coincidences

The momentum distributions of the Ne_2 breakup into Ne^+ <> Ne^0 with the corresponding energy distributions are shown in Supplementary Figure 6. As one can see on the left, apart from the direct, indirect breakup channels and monomer ionization, there is also unwanted background coming from false coincidences. The reason for this is that no selection based on momentum conservation can be applied here, since one particle of the reaction is uncharged (Ne^0) and, thus, is not detected in experiment. Another unwanted source of false coincidences is single ionization of ^{22}Ne, which resides very close to the indirect dissociation channel. The events related to single ionization of ^{22}Ne have been cut by the following momentum conditions [in a.u.]: $-1<p_x<1$ && $-4<p_y<4$ && $-38<p_z<-30$ and not used during further analysis (see the right of Supplementary Figure 6).

The photoelectron momentum distribution in the molecular frame corresponding to the background shows no interference (see Supplementary Figure 7).

We have estimated the background influence on the fringe contrast for the indirect channel. For this, we have plotted the component of the photoelectron momentum that is parallel to the molecular axis ($k_{\parallel}$) for both the indirect breakup channel and the background (see Supplementary Figure 8). The background was chosen by requiring the ion momentum to be within a window of 18-35 a.u. The indirect channel corresponds to the ion momenta in the range of 37-46 a.u. In addition all ion momenta were restricted by $|p_x|<12$ a.u. The volume of the indirect breakup channel in the momentum space was thus by about 0.83 times smaller than that of the background channel. Assuming the constant density of the false coincidences in the momentum space, one get the following estimation for the background signal in the indirect breakup channel caused by false coincidences: 175(red line)*0.83≈145(green lines) counts. This background makes up about a half of the indirect channel background, as seen in the $k_{\parallel}$-projection on the left of Supplementary Figure 8.

The influence of the false coincidences on the **direct** breakup channel is negligible because of its tiny volume in the ion momentum space and large amount of measured events (the ratio of the useful events to false coincidences is very high, which can also be seen in the energy distributions in Supplementary Figure 6).

Supplementary Note 6. The interference fringe contrast

We have calculated the fringe contrast by making use of the projections shown in Supplementary Figure 8. The contrast was found to be 970/330≈2.9 and 580/245≈2.4 for the direct and indirect fragmentation channels, respectively (Supplementary Figure 9).

Supplementary References

1. Demekhin, P. V., Stoychev, S. D., Kuleff, A. I. & Cederbaum, L. S. Exploring Interatomic Coulombic Decay by Free Electron Lasers. *Phys Rev Lett* **107,** 273002 (2011).
2. Wu, J. *et al.* Steering the Nuclear Motion in Singly Ionized Argon Dimers with Mutually Detuned Laser Pulses. *Phys Rev Lett* **110,** 033005 (2013).
3. Paikeday, J. M. & Longstreet, A. Effective potential for e-neon and e-argon scattering by DCS minimization at intermediate energies. *Int. J. Quantum Chem.* **70,** 943–950 (1998).
4. Sann, H. *et al.* Delocalization of a Vacancy across Two Neon Atoms Bound by the van der Waals Force. *Phys Rev Lett* **117,** 263001 (2016).
